# Central nervous system involvement in cardiac amyloidosis: Redefining the heart‐brain axis

**DOI:** 10.1111/eci.70122

**Published:** 2025-09-23

**Authors:** Domenico Mario Giamundo, Giuliano Cassataro, Stefano Ministrini, Simon F. Stämpfli

**Affiliations:** ^1^ Department of Systems Medicine “Tor Vergata” University Rome Italy; ^2^ Department of Medicine and Pulmonology Fondazione Istituto “G. Giglio” Cefalù Italy; ^3^ Department of Health Promotion, Mother and Child Care, Internal Medicine and Medical Specialties (ProMISE) University of Palermo Palermo Italy; ^4^ Center for Molecular Cardiology University of Zurich Schlieren Switzerland; ^5^ Department of Cardiology Luzerner Kantonsspital Lucerne Switzerland

**Keywords:** amyloidosis, cardiac amyloidosis, cognitive decline, ischemic stroke, neurodegeneration, systemic amyloidosis

## Abstract

**Background:**

Amyloidosis is characterised by the extracellular accumulation of misfolded proteins forming amorphous aggregates called amyloid. Cardiac amyloidosis results from myocardial involvement in systemic amyloidosis, leading to impaired heart function. Besides myocardial involvement, cardiac amyloidosis may also directly and indirectly affect the central nervous system.

**Methods:**

This narrative review summarises current evidence about on central nervous system involvement in cardiac amyloidosis and the pathophysiological mechanisms linking heart and brain in the context of this systemic disease.

**Results:**

Although the pathophysiological relationship between cardiac amyloidosis and cognitive decline remains poorly understood, central nervous system involvement likely results from the complex interplay of direct amyloid deposition, cerebrovascular changes, and cardiac dysfunction.

**Conclusion:**

The growing awareness of cognitive impairment in patients with cardiac amyloidosis highlights the need for further research and supports a multidisciplinary approach in the assessment and management of affected individuals.

## Graphical abstract

Central nervous system involvement in cardiac amyloidosis. Amyloidosis is a systemic disease that can directly affect the central nervous system. Furthermore, the amyloid cardiomyopathy can indirectly affect the central nervous system by inducing systemic hypoperfusion and increasing the risk of acute ischaemic stroke. Created with Biorender.com.

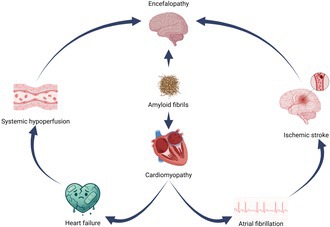

## 1 INTRODUCTION

Amyloidosis is a large, heterogeneous group of progressive, degenerative diseases, characterised by the extracellular accumulation of misfolded proteins, forming amorphous aggregates, named amyloid.^1^ Although some forms almost exclusively affect one organ or system, amyloidosis is generally considered a systemic disease.^2^ Several proteins can generate amyloid, such as serum amyloid A (SAA), transthyretin (TTR), amyloid β, and tau protein. More than 30 proteins have been identified as potentially amyloidogenic, with several different pathophysiologic mechanisms leading to amyloid deposition. For instance, in SAA amyloidosis, the accumulation of pathologic proteins in peripheral tissues is caused by an abnormally increased concentration of the circulating protein, whereas in immunoglobulin light chain amyloidosis (AL) and hereditary ATTR amyloidosis (hATTR), it is caused by an abnormal protein.^3^ Cardiac amyloidosis (CA) is the result of a myocardial involvement in systemic amyloidosis, leading to cardiac dysfunction.^4^ Besides myocardial involvement, CA may directly and indirectly affect the central nervous system (CNS). This narrative review summarises the current evidence about the involvement of CNS in CA and explores the pathophysiological mechanisms linking heart and brain in the context of this systemic disease. This review is based on publications available on PubMed as of April 2025. The literature search included the terms “amyloidosis”, “cardiac amyloidosis”, “AL amyloidosis”, and “ATTR amyloidosis” combined with “central nervous system”, “stroke”, “cognitive decline”, and “dementia”. Additional references were identified by scanning the bibliographies of relevant articles.

## 2 CARDIAC AMYLOIDOSIS: AN OVERVIEW

According to the current knowledge, AL and ATTR amyloidosis account for more than 90% of cases of CA.^5^ However, other amyloidogenic proteins are increasingly identified in CA: Okuda et al. reported that heart failure, electric conductance disorders, and arrhythmias are present in 5%–12% of patients with SAA amyloidosis^6^; Ioannou et al. recently described 13 cases of CA due to accumulation of apolipoprotein AI and 15 cases due to apolipoprotein AIV^7^; and Haslett et al. described one case of CA related to β2‐microglobulin in a non‐dialysed patient.^8^ However, these cases are anecdotal and constitute, at the present time, a small minority of the pathology. Finally, isolated atrial amyloidosis (IAA) is a form of CA localized to the atria, due to the overproduction of atrial natriuretic peptide.^9^ The prevalence of IAA in autopsy studies increases with age, reaching 86%–95% in the ninth decade of life.^10^, ^11^ Age is indeed the most prominent risk factor for this condition, which is proposed by some authors as a potential cause of permanent atrial fibrillation (AF) among elderly people.^12^, ^13^ In systemic amyloidosis, the whole cardiac muscle is affected, including the atria and right ventricle.^14^ However, the mechanical and haemodynamic effects of left ventricle infiltration are the best described. In the left ventricle, amyloid infiltration causes wall thickening and stiffening, regardless of the amyloid type. This leads to diastolic dysfunction, elevated filling pressures, biatrial enlargement, and ultimately heart failure with preserved ejection fraction. While the general pathophysiology is similar, the severity of left ventricular dysfunction depends on the type of the amyloid.^15^

CA can also manifest with conduction abnormalities and arrhythmias such as AF, flutter, atrial tachycardia, or atrioventricular blocks; ventricular arrhythmias can also occur but are less common.^16^

**FIGURE 1:**
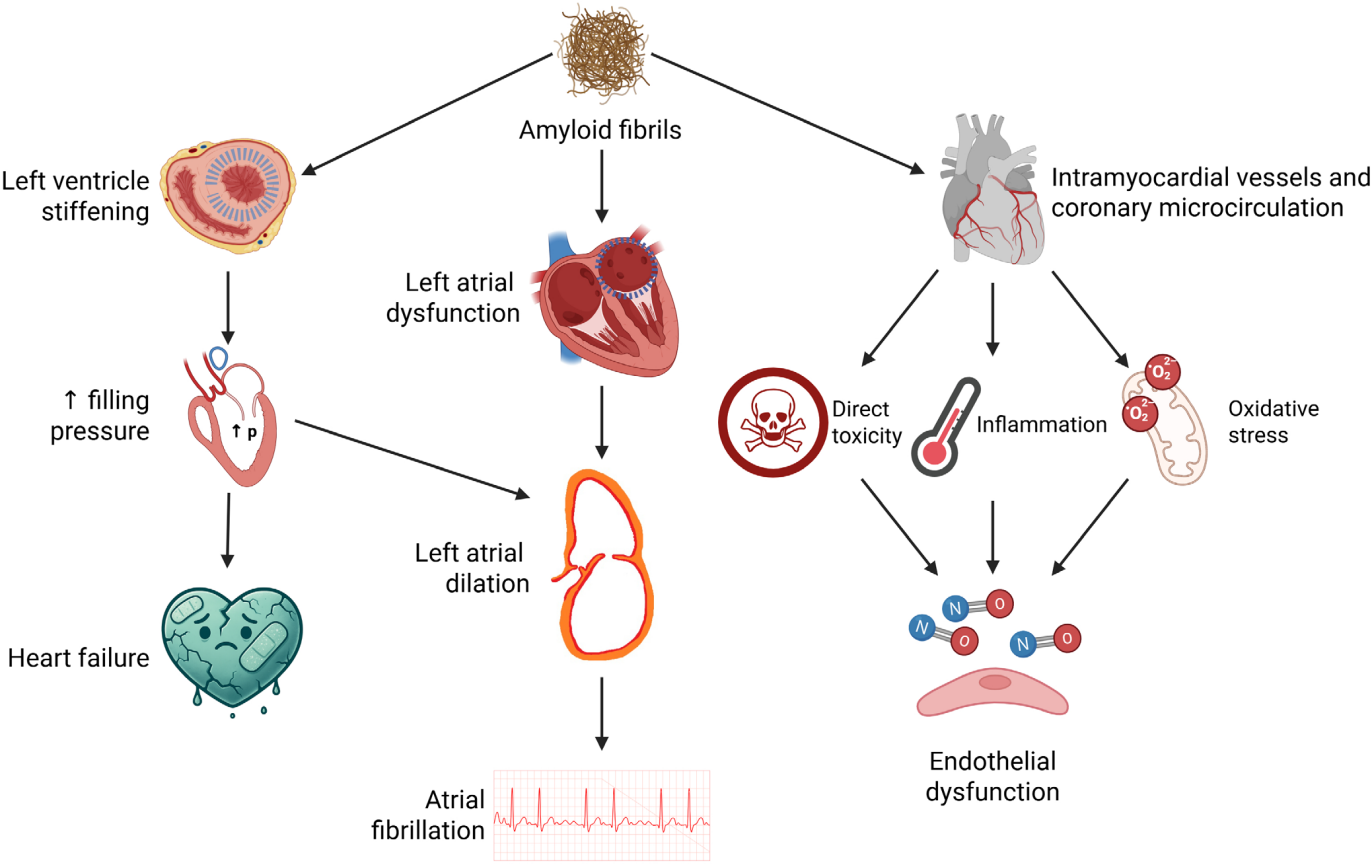
Pathophysiology of cardiac amyloid fibrils infiltration in the heart. Amyloid fibrils accumulation in the heart induces both left ventricular and atrial stiffening. Increased filling pressures as well as volume overload favour atrial dilatation, which is in turn the main anatomical substrate for the onset and perpetuation of atrial fibrillation (AF). Furthermore, the deposition of amyloid in the atria promotes myocardial fibrosis, directly predisposing to AF and hampering atrial contraction, independent of atrial size. Atrial mechanical dysfunction and most likely endocardial dysfunction favour blood stasis and thrombus formation, thus increasing the risk of stroke. Cardiac amyloidosis is characterised by a reduced coronary flow reserve due to endothelial dysfunction. Amyloid infiltrates intramyocardial vessels, leading to a reduction in myocardial perfusion, possibly contributing to the myocardial damage. The microvascular dysfunction is determined by capillary rarefaction, extrinsic compression of the microvasculature, and endothelial dysfunction. Capillary rarefaction is due to the combined effect of oxidative stress, inflammation, and reduced angiogenesis. Amyloid deposition induces the production of oxygen free radicals and reduces the antioxidant activity. Amyloid fibrils act as damage‐associated molecular patterns recognised by resident macrophages, triggering the release of pro‐inflammatory cytokines. Beyond the above‐described mechanisms, a toxic effect of amyloid and its precursor proteins, causing a direct damage to the endothelium, has been proposed as a potential cause of endothelial dysfunction. Created with Biorender.com and DALL·E3.

A comprehensive overview of the pathophysiological mechanisms underlying CA is summarised in Figure 1.

AL amyloidosis results from a clonal proliferative disorder of plasma cells, leading to excessive production of light chains (LCs). LCs are deposited as amyloid fibrils in multiple organs, including the heart in 50% of cases.^17^ Cardiac dysfunction in AL amyloidosis is not only due to the mechanical effect of intramyocardial amyloid deposition, but also to the cardiotoxic effect of light chain aggregates.^18^ Indeed, LCs can directly affect cardiomyocyte function by increasing oxidative stress, inducing mitochondrial dysfunction and impairing intracellular calcium handling, eventually resulting in contractile dysfunction, as confirmed by in vivo preclinical studies.^18^, ^19^, ^20^, ^21^

Awareness of cardiac ATTR amyloidosis has increased significantly in recent years, making it the most frequently diagnosed form of CA.^22^ It is caused by the deposition of misfolded ATTR, a liver‐derived plasma protein responsible for transporting lipophilic substances such as retinol and thyroid hormones.^23^ There are two forms of ATTR amyloidosis, each with distinct causes and clinical features: a hereditary form (hATTR), caused by mutations in the TTR gene leading to a defective TTR protein, and a wild‐type form (wtATTR) of unknown cause, which primarily affects older male individuals. Hereditary ATTR follows an autosomal dominant inheritance pattern with variable penetrance. The onset of the disease is commonly around the fifth decade of life but varies depending on the present mutation.^24^ TTR is stable and functionally active in a tetrameric form. In hATTR, mutations in the TTR gene lead to structural instability of the protein, resulting in dissociation into dimers or monomers, which subsequently misfold and aggregate.^25^ The steps leading from protein destabilization to misfolding and amyloid formation are still incompletely understood. However, one can hypothesise that TTR monomers can cross the endothelium and infiltrate the perivascular interstitial space, where they misfold and form the typical fibrillary aggregates. More than 120 mutations of the TTR gene have been described. While most variants primarily cause a neurological phenotype, several mutations are associated with significant or predominant cardiac involvement.^26^, ^27^ For instance, Val30Met, the most common variant worldwide, can present with two different phenotypes: an early onset phenotype (before the sixth decade of life), mainly characterised by autonomic neuropathy, and a late onset, with a mixed phenotype, mainly characterised by cardiomyopathy and sensory‐motor neuropathy.^28^ In contrast, Ile68Leu, the most common mutation in Italy,^29^ is associated with a prevalent cardiac and musculoskeletal involvement, clinically indistinguishable from wtATTR.^28^ Wild‐type ATTR is characterised by a normal primary structure of the protein; therefore, the cause of dissociation of the TTR tetramer is unknown. Wild‐type ATTR predominantly affects males and has a late onset, usually after the seventh decade of life.^30^, ^31^ Despite being a systemic disease, cardiac dysfunction is generally the main clinical feature. However, in addition to primary myocardial dysfunction, epidemiological studies highlighted an association between wtATTR and aortic stenosis, suggesting a potential involvement of valve structures, contributing to the initiation or progression of aortic valve stenosis.^32^, ^33^, ^34^ However, this association could also be also due to common risk factors, such as old age, and therefore further mechanistic studies are necessary to confirm this hypothesis. Among extra‐cardiac manifestations, musculoskeletal disorders such as bilateral carpal tunnel syndrome, lumbar spinal stenosis, and biceps tendon rupture are common and often precede cardiac symptoms by more than 10 years.^35^, ^36^ An incidental clonal dyscrasia of plasma cells is found in up to 25% of patients with wtATTR, highlighting the critical need to exclude an AL amyloidosis by histological analysis.^37^ Although exceedingly rare, the coexistence of ATTR and AL amyloidosis in the same subject is also possible.^38^

Therapeutic options for patients with CA have significantly progressed over the past decade, from symptomatic and supportive treatment to specific, disease‐modifying therapies.^39^, ^40^ The emergence of novel therapeutic strategies has accelerated both clinical and translational research in this field, driving a progressive refinement of diagnostic workflows and advancing the understanding of the underlying pathophysiology.^24^ In AL amyloidosis, the treatment aims at eradicating the plasma cell clonal population, by immune‐chemotherapy and/or autologous bone marrow transplantation. The recent introduction of a highly effective plasma‐cell‐directed therapy, based on the combination of the proteasome inhibitor bortezomib, with the anti‐CD38 monoclonal antibody daratumumab, dexamethasone, and cyclophosphamide (Dara‐CyBorD), yielded an unprecedented rate of good haematological response close to 80%.^41^ The treatment of TTR amyloidosis has been revolutionized by the introduction of TTR tetramer stabilizers such as tafamidis and acoramidis as well as TTR gene silencers such as patisiran, vutrisiran, and eplontersen. The former act by stabilizing the transthyretin tetramer,^42^ while the latter use small interfering RNA (siRNA) or antisense oligonucleotides to block the translation of TTR messenger RNA.^43^ More futuristic perspectives are already under investigation in clinical trials, including an in vivo gene‐editing approach using clustered regularly interspaced short palindromic repeats and associated Cas9 endonuclease (CRISPR‐Cas9) technology and a human monoclonal antibody against TTR amyloid.^44^, ^45^

## 3 CARDIAC AMYLOIDOSIS AND ACUTE ISCHEMIC STROKE

AF is a very common complication of CA, with an estimated prevalence of 44%, compared to the 1% in the general population (9% in subjects older than 80 years).^46^, ^47^ The highest prevalence has been reported among patients with wtATTR, probably due to older age and a higher burden of comorbidities.^48^ Multiple mechanisms predispose to supra‐ventricular arrhythmias in CA: (1) amyloid deposition in the atrial wall induces a structural remodelling, thus predisposing to electrical disturbances, such as intra‐atrial re‐entry^49^; (2) bi‐atrial dilation further promotes AF and other atrial arrhythmias.^50^, ^51^, ^52^ Masri et al. conducted a retrospective analysis of 84 patients with TTR amyloidosis to determine the prevalence of AF over a 14‐year follow‐up and found that around 50% had AF already before the diagnosis of CA, with an additional 25% occurring after the diagnosis.^53^ These findings suggest that atrial electrical disturbances occur early in the course of the disease, and that greater emphasis should be placed on the early detection of ATTR. A recent cross‐sectional study estimated that 4% of patients with AF undergoing cMR imaging prior to catheter ablation have suggestive signs of CA; of them, 38% were symptomatic with ischaemic stroke.^54^ Further longitudinal studies are warranted to determine which patients with AF should be screened for CA.

**FIGURE 2:**
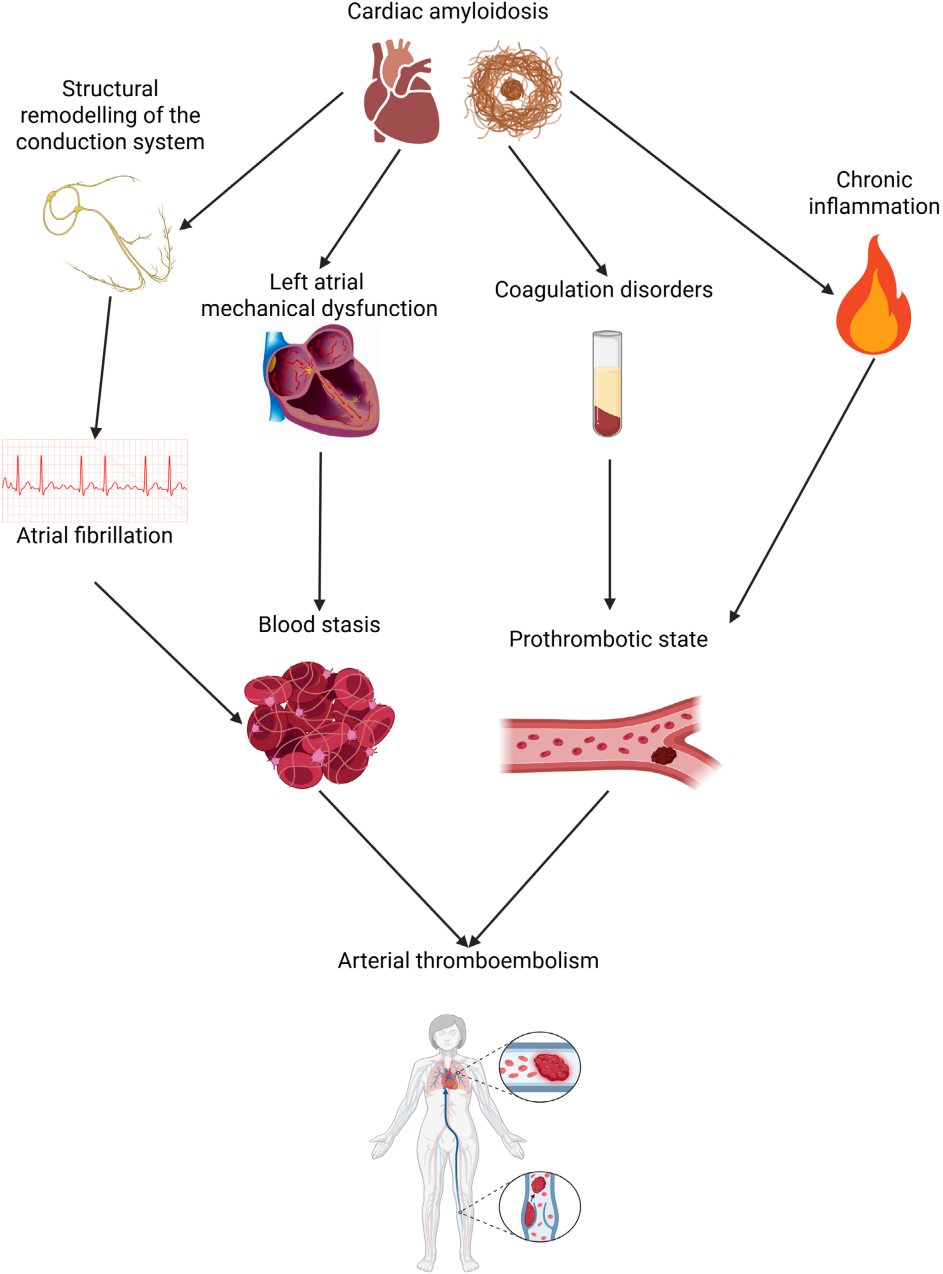
Pathological mechanisms leading to thromboembolic events in cardiac amyloidosis. Cardiac amyloidosis is characterised by an extraordinarily elevated risk of systemic embolism, due to the co‐existence of three factors: atrial arrhythmia, left atrial stiffness, and a pro‐thrombotic state. Created with Biorender.com.

Several studies demonstrate that the presence of AF in patients with CA carries a high risk of stroke and systemic embolism.^55^ Therefore, recent guidelines of the European Society of Cardiology suggest that anticoagulant therapy should be administered regardless of the CHA_2_DS_2_‐Vasc score in patients with CA and concomitant AF.^56^ However, AF is not the sole cause of thromboembolic events in CA. The mechanical dysfunction of the atrium, secondary to amyloid infiltration and increased filling pressure, impairs atrial contractility and induces blood stasis as well as endothelial damage, thus promoting intra‐atrial thrombosis irrespective of the presence of AF.^57^, ^58^, ^59^ An autopsy series of 54 patients with CA revealed intracardiac thrombi in 14 patients, despite only 9 patients having a known history of AF.^60^ Consistently, an imaging study in 80 patients with CA yielded intracardial thrombi in 27% of them. Notably, intra‐atrial thrombi were also observed in around 20% of patients without documented AF.^55^ Data from a large multicenter registry, investigating 1191 patients with TTR amyloidosis, revealed an incidence rate of thromboembolic events of 1.3 per 100 patients‐year among patients in sinus rhythm who were not on anticoagulant therapy, of 1.7 in patients with AF on anticoagulant therapy, and 4.8 in patients with AF without anticoagulant therapy.^61^ The occurrence of thrombosis in CA patients with sinus rhythm is intriguing: several mechanisms have been proposed to explain this phenomenon, including atrial dysfunction due to amyloid infiltration, among others.^62^ CA itself could be a pro‐thrombotic status: coagulation disorders have been described in AL amyloidosis, likely due to concomitant nephrotic syndrome, reduced mobilization and chemotherapy.^63^ Furthermore, aggregated proteins containing cross‐β structures, such as TTR amyloid, can activate the coagulation cascade and form an amyloid‐fibrin clot that is resistant to degradation by plasmin.^64^, ^65^, ^66^ By studying extracellular vesicles, Napolitano et al. observed an immune‐mediated endothelial damage in a patient with ATTR and stroke suggesting that inflammation plays a role in the hypercoagulability of CA patients.^67^ As previously demonstrated, several species of extracellular vesicles can have procoagulant properties due to the expression of negatively charged membrane phospholipids (e.g., phosphatidylserine) or coagulation factor III.^68^, ^69^, ^70^ Recent in vitro evidence suggest that tafamidis could have an anti‐thrombotic effect, irrespective of its TTR stabilizing activity.^71^ Despite these observations, a direct prothrombotic effect of TTR amyloid has not yet been demonstrated (Figure 2).

## 4 CENTRAL NERVOUS SYSTEM INVOLVEMENT IN SYSTEMIC AMYLOIDOSIS

Alzheimer's disease (AD) is the most well‐known and studied type of CNS amyloidosis, characterised by the deposition of β‐amyloid plaques in the brain.^72^ Although AD mainly involves the CNS, an association with other cardiovascular disorders, such as systemic arterial hypertension and heart failure, has been hypothesised.^73^ Cerebral amyloid angiopathy (CAA) is another common form of CNS amyloidosis, caused by β‐amyloid deposits around small cerebral arteries, both cortical and leptomeningeal, leading to vessel stiffening, fragility, and an increased risk of haemorrhagic stroke. CAA is also a common cause of cognitive decline and can be associated with AD and other systemic amyloidoses.^74^ In CAA, amyloid plaques are mainly constituted by Aβ_40_, a fragment of the amyloid precursor protein (APP) similar to Aβ_42_, characterising AD. Indeed, CAA and AD can be described as two extremes of a spectrum, with the Aβ40:Aβ42 ratio deciding the localisation of the amyloid deposits.^75^ Like AD, CAA is usually a sporadic, age‐related disease, although hereditary forms of the disease have been described, like Dutch‐type CAA, caused by mutations in the APP gene and characterised by early onset and a malignant course.^76^ CAA can cause cognitive impairment before the occurrence of overt cerebrovascular events, indicating that small vessel pathology may play an independent role in cognitive decline.^77^, ^78^

In the context of systemic amyloidosis, peripheral neuropathy (PN) is the most common and best described neurological complication. Both AL and TTR amyloidosis can cause peripheral neuropathy, and this symptom usually occurs usually early during the disease course, with a higher prevalence in TTR amyloidosis, particularly hATTR.^79^ Sensory symptoms, like loss of temperature sense, hypoesthesia, or neuropathic pain, usually precede motor symptoms and autonomic dysfunction.^80^ When present, autonomic dysfunction is a strongly disabling manifestation. Gastrointestinal symptoms are usually the earliest and most common presentation of amyloid autonomic dysfunction: diarrhoea is the typical symptom, which distinguishes amyloid PN from other autonomic dysfunctions, more likely characterised by constipation. Gastrointestinal dysfunction can be further complicated by nausea, vomiting, abdominal pain, and ultimately weight loss. Later symptoms are erectile dysfunction, heat intolerance, decreased sweating, and orthostatic hypotension. Weight loss and orthostatic hypotension may significantly complicate the management of patients with co‐existing heart failure because they can worsen cachexia and increase the risk of adverse reactions to pharmacological treatment.^81^

CNS involvement has been described in patients with hATTR.^82^, ^83^ This involvement was initially described as a form of CAA, but TTR‐related cerebral amyloidosis was later recognised as having peculiar neuropathological features.^84^ Indeed, neuropathological studies of patients with hATTR carrying the Val30Met mutation described a unique pattern of amyloid deposits in the brain, following a specific pattern of progression: the process begins around the vessels, first involving the leptomeninges and subarachnoid meningeal vessels, then extending to the perforating cortical vessels and the subpial space, and finally affecting the subependymal region and the vessels of the basal ganglia. The brainstem and spinal cord are also affected at early stages. However, amyloid deposition appears to be largely confined to superficial structures, with limited deep parenchymal involvement.^83^, ^85^ Similar neuropathological findings, previously labelled as oculoleptomeningeal amyloidosis, have also been also described in single‐case reports of patients with other TTR mutations, such as Asp18Gly, Ala25Thr, Val30Gly, Tyr69His, Tyr114Cys, and Gly47Arg,^86^, ^87^, ^88^ suggesting that the CNS involvement in hATTR is not specific to a few mutations. These findings are unlikely innocent bystanders, but are associated with neurological symptoms, suggesting a mechanistic role of amyloid deposition in the neurological involvement of hATTR. Several case series describe focal neurological symptoms (FNS) in 20%–30% of patients with Val30Met mutation, treated with liver transplantation. FNS represent a late manifestation of hATTR, occurring around 15 years after the onset of the peripheral neuropathy and 10 years after liver transplantation.^85^, ^89^ Described FNS include a wide range of acute neurological disorders, such as seizures, migraine with aura, transient ischaemic episodes, ischaemic stroke, and intra‐parenchymal haemorrhage.^84^, ^85^, ^88^, ^89^, ^90^, ^91^ Neuropathological features of AD, CAA, and leptomeningeal amyloidosis are summarised in Table 1.

**TABLE 1:** Neuropathological features of different forms of brain amyloidosis. Brain amyloidosis often presents overlapping features, and a definitive differential diagnosis is only possible by a molecular characterisation of the amyloid protein on histological preparations.

	Alzheimer's disease	Cerebral amyloid angiopathy	Transthyretin amyloidosis
Cortical atrophy	✓	✘	✘
Intracellular tangles	✓	✘	✘
Peri‐vascular amyloidosis	✘	✓	✓
Capillary amyloidosis	✘	✓	✘
Involvement of meningeal vessels	✘	✓	✓
Patchy pattern	✓	✓	✘
Spatial–temporal progression pattern	✘	✘	✓
Micro‐bleedings	✘	✓	✓
Ischemic stroke and micro‐infarctions	✘	✘	✓
White matter disease	✘	✓	✘
Basal ganglia involvement	✘	✘	✓
Hippocampal and amygdala involvement	✓	✘	✘

Beyond acute FNS, hATTR has been associated with cognitive decline and other neuro‐psychiatric symptoms. This association was first described by Petersen et al.^92^ in a family of Val30Gly mutation carriers. Symptomatic carriers had predominant central manifestations, including progressive dementia, seizures, and visual impairment, without peripheral neuropathy, underscoring the clinically relevant role of primary CNS involvement in hATTR. More recently, an observational study on 340 carriers of the Val30Met mutation revealed an age‐dependent, measurable impairment across multiple cognitive domains, such as attention and executive function, in symptomatic subjects compared to the asymptomatic ones.^93^ These findings reinforce the need for systematic cognitive evaluation in patients with hATTR, particularly in those with long disease duration or advanced disease. Whether patients with wtATTR can have a CNS involvement, and especially an accelerated cognitive decline, is still an unaddressed question. Should such involvement be demonstrated, ATTR amyloidosis could represent an emerging, highly prevalent cause of dementia among the elderly people. In this regard, a recent consensus document highlighted the importance of a comprehensive geriatric assessment in older patients with ATTR amyloidosis, including a cognitive function alongside other parameters.^94^

Di Paolantonio et al.^95^ described the case of two siblings who experienced cognitive decline 30 years after liver transplantation for hATTR, suggesting that brain amyloid deposits can become symptomatic after several years and even after the removal of the main source of the pathologic protein. This observation poses a significant challenge for long‐term management. As described above, novel therapies for ATTR amyloidosis focus on silencing the expression of the TTR gene or promoting the clearance of misfolded TTR aggregates through monoclonal antibodies.^45^, ^96^ These approaches have shown promising results in halting or reversing cardiac involvement, significantly improving both morbidity and survival in affected patients. However, despite these benefits, their efficacy on CNS manifestations remains unclear. These uncertainties highlight a critical knowledge gap with possible therapeutic drawbacks: while current interventions are reshaping the management of systemic and cardiac amyloidosis, they may fall short in preventing neurologic and cognitive complications, especially in patients with long‐standing disease. Interestingly, high circulating levels of tetrameric TTR have been suggested to exert potentially protective effects in AD, as recently reviewed by Corino et al.^97^ These findings raise the possibility that strong suppression of TTR expression through gene‐silencing therapies or complete TTR knockout via CRISPR‐Cas may have unintended consequences, such as accelerating cognitive decline in patients with ATTR amyloidosis. While CNS involvement in ATTR amyloidosis remains underdiagnosed, advanced imaging techniques are becoming increasingly valuable for identifying cerebral involvement. Sekijima et al.^84^ described a unique pattern of cerebral radiotracer accumulation using amyloid PET imaging. More recently, brain MRI has also emerged as a useful complementary tool, capable of detecting specific signal abnormalities, which help differentiate ATTR‐related CNS involvement from other neurodegenerative diseases like AD.^98^ Beyond ATTR amyloidosis, CNS involvement has been observed in other forms of systemic amyloidosis. Hereditary gelsolin amyloidosis (HGA) is a rare, autosomal dominant condition caused by mutations of gelsolin, a cytosolic protein, mainly involved in cytoskeleton remodelling. Typical manifestations of HGA are peripheral neuropathy, with a prevalent involvement of cranial nerves, dermatological findings, and corneal dystrophy.^99^ However, CNS involvement has been described too, with a leptomeningeal pattern of amyloid deposition. Usual features of CNS involvement in HGA are degeneration of the posterior columns of the spinal cord and atypical neurocognitive and neuropsychiatric symptoms.^99^, ^100^ Interestingly, also cardiovascular disorders have also been associated with HGA, especially conduction abnormalities such as atrio‐ventricular blocks and sick sinus syndrome.^101^, ^102^

## 5 CONCLUSIONS

Although cardiac involvement is the primary determinant of mortality and disability in patients with CA, due to the systemic nature of the disease, several other organs are affected, including the CNS.

CNS can be directly affected by the deposition of misfolded amyloid proteins, typically presenting as CAA or leptomeningeal amyloidosis. In both conditions, arterial stiffening and microvascular dysfunction may impair cerebral blood flow, further contributing to cognitive impairment.

In addition to the direct effects of amyloid deposition in the CNS, neurological symptoms can result from cardiovascular dysfunction and its associated complications, such as AF and cerebrovascular events. However, the burden of neurological disability due to cerebrovascular diseases and cardiac impairment in patients with CA has not been estimated yet. This aspect is particularly relevant in subjects with wtATTR, which currently constitutes the most common form of CA and could represent a neglected and underdiagnosed cause of neurocognitive decline in the elderly population. In general, a thorough clinical, radiological, and pathological characterization of the neurological involvement in patients with CA is a current scientific pitfall, warranting future research efforts.

In summary, although the pathophysiological mechanisms linking amyloidosis and cognitive decline are not yet fully understood, the interplay between systemic amyloid deposition, cerebrovascular changes, and cognitive function highlights the importance of a multidisciplinary approach to the assessment and management of affected patients. Growing awareness of potential CNS involvement in systemic amyloidosis suggests that subtle cognitive deficits – often affecting non‐typical domains such as attention and executive function – may occur even in the absence of overt neurological symptoms. This emerging evidence supports routine cognitive screening in patients with CA, particularly in older adults.

## AUTHOR CONTRIBUTIONS

D.M.G. and G.C. wrote the initial draft and prepared figures/tables; S.M. finalised the manuscript; S.F.S. conceptualised the review, supervised the writing, and provided funding.

## FUNDING INFORMATION

This work has been supported by the Pfizer Competitive Research Grant for Switzerland and the GAMBIT Gemeinnützige Stiftung.

## CONFLICT OF INTEREST STATEMENT

PD Dr. Stämpfli is has received travel grants, speaker fees, consulting fees, or proctoring fees from Abbott, Alnylam, Amgen, AstraZeneca, Bristol‐Myers Squibb, Daiichi‐Sankyo, Edwards, Polares Medical, Pfizer, and Takeda. Dr. Ministrini has received financial support from the Swiss Heart Foundation, outside this work. All the remaining authors have no competing interest to declare.

## Data Availability

No original data was generated for this paper.
